# A highly efficient rod-like-CeO_2_-supported palladium catalyst for the oxidative carbonylation of glycerol to glycerol carbonate

**DOI:** 10.1039/d1ra02187g

**Published:** 2021-05-10

**Authors:** Ziyan Wang, Shuo Guo, Zhimiao Wang, Fang Li, Wei Xue, Yanji Wang

**Affiliations:** Hebei Provincial Key Laboratory of Green Chemical Technology and High Efficient Energy Saving, School of Chemical Engineering and Technology, Hebei University of Technology Tianjin 300130 China weixue@hebut.edu.cn wangzhimiao@hebut.edu.cn; Tianjin Key Laboratory of Chemical Process Safety Tianjin 300130 China; Hebei Industrial Technology Research Institute of Green Chemical Industry Huanghua 061100 Hebei China

## Abstract

A rod-like-CeO_2_-supported Pd catalyst (Pd/CeO_2_-r) was prepared using two-step hydrothermal impregnation and used in the oxidative carbonylation of glycerol to produce glycerol carbonate. The characterization results showed that the Pd was highly dispersed on the surface of the CeO_2_-r, and metallic Pd was the main species in the catalyst. The Pd/CeO_2_-r exhibited good catalytic performance for the oxidative carbonylation of glycerol. Under optimized reaction conditions, the glycerol conversion and glycerol carbonate selectivity were 93% and 98%, respectively, and turnover frequency was 1240 h^−1^. However, because of the leaching of Pd and the growth of Pd particles, the catalyst was gradually deactivated throughout reuse.

## Abstract 2

Pd/CeO_2_-r was prepared by a two-step hydrothermal-impregnation method for oxidative carbonylation of glycerol. It exhibited high activity, and glycerol conversion was 93% and glycerol carbonate selectivity was 98% with a TOF of 1240 h^−1^ under optimized conditions.

## Graphical abstract

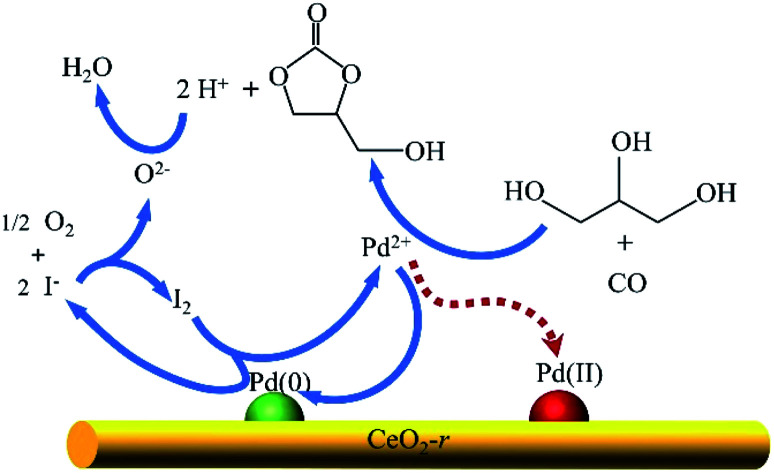

## 1 Introduction

**Scheme 1:**
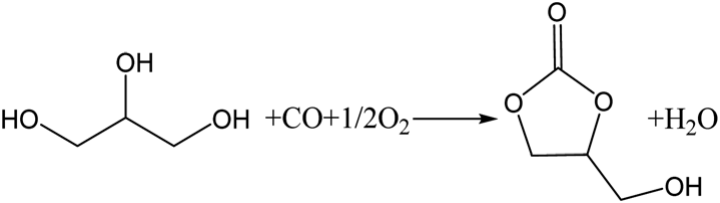
Chemical equation for the oxidative carbonylation of GL to GLC.

In recent years, concerns about global warming have resulted in increased interest in the biodiesel industry as a source of renewable energy.^1^ However, during the manufacture of biodiesel, a large quantity of glycerol (GL) is produced as a by-product.^2^ For every 10 tons of biodiesel produced, approximately 1 ton of GL is produced. As biodiesel is produced on a large scale, the use of GL has become an important factor affecting the development of the biodiesel industry. GL can be converted into valuable chemicals by reaction, such as acrolein,^3^ glycerol ether,^4^ 1,2-propanediol,^5^ lactic acid,^6,7^ and glycerol carbonate (GLC).^8^ GLC is an important derivative of GL with a high boiling point, high biodegradability, low flammability, and low toxicity. It can be used as a green solvent in the fields of paint, cosmetics, medicine, and lithium batteries. It can also be used as a raw material in the synthesis of surfactants, agrochemicals, and polymers.^9,10^ Therefore, much attention has been given to the synthesis of GLC.^11^ There are several methods for the synthesis of GLC from GL, including phosgenation,^2^ transesterification,^12,13^ urea alcoholysis,^14,15^ and the oxidative carbonylation of GL.^16^ The oxidative carbonylation of glycerol (OCG) is a highly efficient and attractive approach for obtaining GLC because of the high availability and low cost of CO and O_2_, and the high atom economy of the process.^17^ The reaction equation is shown in Scheme 1.

The catalysts used for the OCG reaction include homogeneous and heterogeneous catalysts. Hu *et al.*^17^ explored a homogeneous PdCl_2_(phen)/KI (phen = 1,10-phenanthroline) catalyst system for the OCG reaction. GL conversion was 92%, GLC selectivity was 99%, and the turnover frequency (TOF) reached 184 h^−1^. Casiello *et al.* studied the homogeneous OCG reaction using CuCl_2_ as a catalyst and pyridine as a co-catalyst.^16^ Excellent conversions (>92%) and selectivities (>93%) were obtained, and it was found that Cu^+^ is the intermediate species in the catalytic cycle. Because of the inherent disadvantages of homogeneous catalysts, such as the difficulty in separation and recovery of catalysts, heterogeneous catalysts have been attracting increasing attention. Hu *et al.*^18^ developed a zeolite-Y-confined Pd catalyst (PdCl_2_(phen)@Y) for the OCG reaction. This heterogeneous catalyst exhibited a high activity, with a reasonable GL conversion of 95%, GLC selectivity of 98%, and a TOF of 317 h^−1^. The catalyst could be reused five times without a significant decrease of the catalytic activity. Wang *et al.*^19^ studied a commercially available Pd/C catalyst for GLC synthesis *via* the OCG reaction with the aid of NaI. Under the optimum conditions, a TOF of 900 h^−1^ was obtained. Lei *et al.*^8^ reported Pd@PQP-NHC as a recyclable catalyst for the OCG reaction. It could afford 96% conversion of GL with a high TOF of 1901 h^−1^.

The oxidative carbonylation reaction involves electron transfer. Pd compounds are usually used as catalysts with various co-catalysts. Ceria, widely used in various oxidation reactions,^20,21^ exhibits a good promotion effect for Pd-catalyzed oxidative carbonylation because of its high oxygen adsorption capacity and good oxygen storage and release (Ce^3+^/Ce^4+^) performance. In our previous work,^22^ a Pd–Ce–O/SiO_2_ catalyst was prepared using a microemulsion method. The catalyst was used for the oxidative carbonylation of phenol to synthesize diphenyl carbonate (DPC). It was found that Ce^4+^ entered the PdO crystal lattice, which meant that the electrons of the deactivated Pd could easily be transferred to the Ce. Therefore, the Pd–Ce–O/SiO_2_ catalyst was easily regenerated and exhibited good performance. Yuan^23^ and Fu *et al.*^24^ studied the oxidative carbonylation of phenol to produce DPC over a Pd catalyst supported on CeO_2_ nanotubes. This catalyst exhibited excellent performance. They also found that the morphology of the CeO_2_ had a significant effect on the performance of the catalyst. CeO_2_ can also be used as a catalyst for the carbonylation of GL with CO_2_ to produce GLC, as described in ref. 25. The GLC yield in this study was up to 78.9%, and the CeO_2_ catalyst could be regenerated simply by calcination after 5 cycles.

CeO_2_ is very important in the oxidative carbonylation reaction. However, as far as we know, CeO_2_ has not been used in the OCG reaction to produce GLC. In this paper, a rod-like-CeO_2_-supported palladium catalyst (Pd/CeO_2_-r) was prepared, and its performance in the OCG reaction was evaluated. The Pd/CeO_2_-r exhibited excellent catalytic performance with GL conversion of 93% and GLC selectivity of 98%. We also discuss the stability and catalytic mechanism of the Pd/CeO_2_-r catalyst.

## 2 Experimental

### 2.1 Experimental section chemicals

Polyethylene oxide–polypropylene oxide–polyethylene oxide block copolymer (P123) and glycerol were purchased from Sigma-Aldrich. CeCl_3_·7H_2_O, aqueous ammonia (28 wt%), absolute ethanol, PdCl_2_, and NaBH_4_ were purchased from Sinopharm Chemical Reagent Co., Ltd. NaI and dimethyl acetamide were purchased from Macklin. All chemicals were used without further purification.

### 2.2 Catalyst preparation

P123 (17.4 g) was dissolved in a mixture of absolute ethanol (60 mL) and deionized water (60 mL) with ultrasonic treatment in a beaker. CeCl_3_·7H_2_O (5.58 g) was then added to the solution. With vigorous stirring, NH_3_·H_2_O (28 wt%) was added dropwise until the pH value of the solution was 10. The solution was then stirred for another 30 min. The resulting suspension was rapidly transferred into a polytetrafluoroethylene (PTFE)-lined stainless steel autoclave with a capacity of 200 mL, and hydrothermally treated at 160 °C for 72 h. After cooling to room temperature, the solid was separated by centrifugation and washed with water and ethanol until the filtrate was neutral. The resulting solid was then dried at 60 °C and calcined at 500 °C for 4 h with a heating rate of 5 °C min^−1^. Finally, a light-yellow powder, the rod-like CeO_2_, was obtained, and was denoted by CeO_2_-r.

PdCl_2_ (0.018 g) was dissolved in a mixture of deionized water (10 mL) and NH_3_·H_2_O (28 wt%, 5 mL) with ultrasonic treatment. CeO_2_-r (0.36 g) was dispersed in deionized water (10 mL). Then the two mixtures were mixed together and stirred for 2 h. Next, NaBH_4_ solution (0.010 g NaBH_4_ dissolved in 10 mL H_2_O) was added dropwise into the mixture, which was stirred for 24 h. The solid was filtered and washed with ethanol and dried in a vacuum at 80 °C. Finally, the solid was calcined in a tube furnace at 300 °C under flowing nitrogen gas. The obtained catalyst was denoted by Pd/CeO_2_-r and had a nominal Pd loading of 3.0 wt%.

### 2.3 Catalyst characterization

Scanning electron microscope (SEM) of FEI Nova Nano SEM 450 type was carried out to observe the morphology of the catalyst particles. Transmission electron microscope (TEM) and selected area electron diffraction (SAED) were observed with PHILIPS TECNOL 20. X-ray diffraction (XRD) patterns were recorded on a Bruker D8 FOCUS X-ray diffractometer with Cu Kα radiation (40 kV) and a secondary beam graphite monochromator (SS/DS = 1°, RS 0.15 mm, counter SC) at the scanning 2*θ* range of 5°–90°. The specific surface areas of the samples were calculated by BET equation using N_2_ adsorption–desorption technique with a Micromeritics ASAP 2020M + C porosity analyzer. Thermo Scientific Escalab 250 Xi photoelectron spectrometer (14.6 kV, 200 W) with Al Kα (1486.6 eV) were used for X-ray photoelectron spectroscopy (XPS) and the number of scanning times was 20. The correction was performed with C 1s (284.8 eV). Pd content in the catalyst was determined by a Thermo Scientific iCAP 7400 Inductively Coupled Plasma-Optical Emission Spectroscopy (ICP-OES).

### 2.4 Catalyst evaluation

The catalyst activity was evaluated in a stainless-steel autoclave of Amtech® Slurry Phase Reactor Systems with an inner volume of 50 mL. In a typical experiment, glycerol, Pd/CeO_2_-r catalyst, co-catalyst (NaI) and solvent (dimethyl acetamide, DMA) were added into the autoclave. Then the autoclave was sealed and pressurized with a mixture of O_2_ (1.7 MPa) and CO (3.3 MPa) and the temperature was heated to the desired value. After a period of time, the autoclave was cooled to room temperature and vented. The catalyst was separated by centrifugation, and the liquid phase was analyzed by gas chromatograph.

The reaction products were identified quantitatively using an Agilent 7890B gas chromatograph with a KB-Wax capillary column (30 m × 0.32 mm × 0.50 μm) and a flame ionization detector. Column temperature started at 80 °C and was increased to 260 °C at a rate of 30 °C min^−1^. Nitrogen was used as the carrier gas. The quantitative analysis of the reaction products were carried out using *n*-butanol as internal standard.

After the OCG reaction, the Pd/CeO_2_-r catalyst was recovered by centrifugation, washed thoroughly with ethanol, and dried at 80 °C for 12 h. It was then reused directly in OCG reaction.

## 3 Results and discussion

### 3.1 Catalyst characterization

**Fig. 1:**
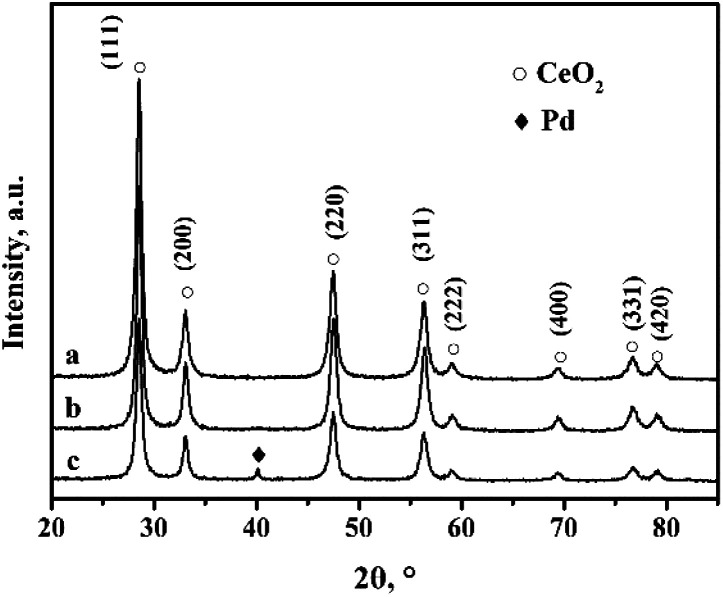
XRD patterns of (a) CeO_2_-r, (b) Pd/CeO_2_-r, and (c) recovered Pd/CeO_2_-r.

Fig. 1 shows the XRD (X-ray diffraction) patterns of the CeO_2_-r and Pd/CeO_2_-r. There are no obvious differences between the diffraction peak positions and strengths in the patterns of the two samples. The peaks at 2*θ* = 28.51°, 33.10°, 47.62°, 56.53°, 59.07°, 69.45°, 76.74°, and 79.21° are attributed to the (111), (200), (220), (311), (222), (400), (331), and (420) crystal planes of CeO_2_ with a cubic structure (JCPDS65-5923). Peaks corresponding to Pd metal or Pd compounds were not observed in the XRD patterns of the Pd/CeO_2_-r, which may be because a small amount of Pd species was highly dispersed on the CeO_2_.^26,27^

**Fig. 2:**
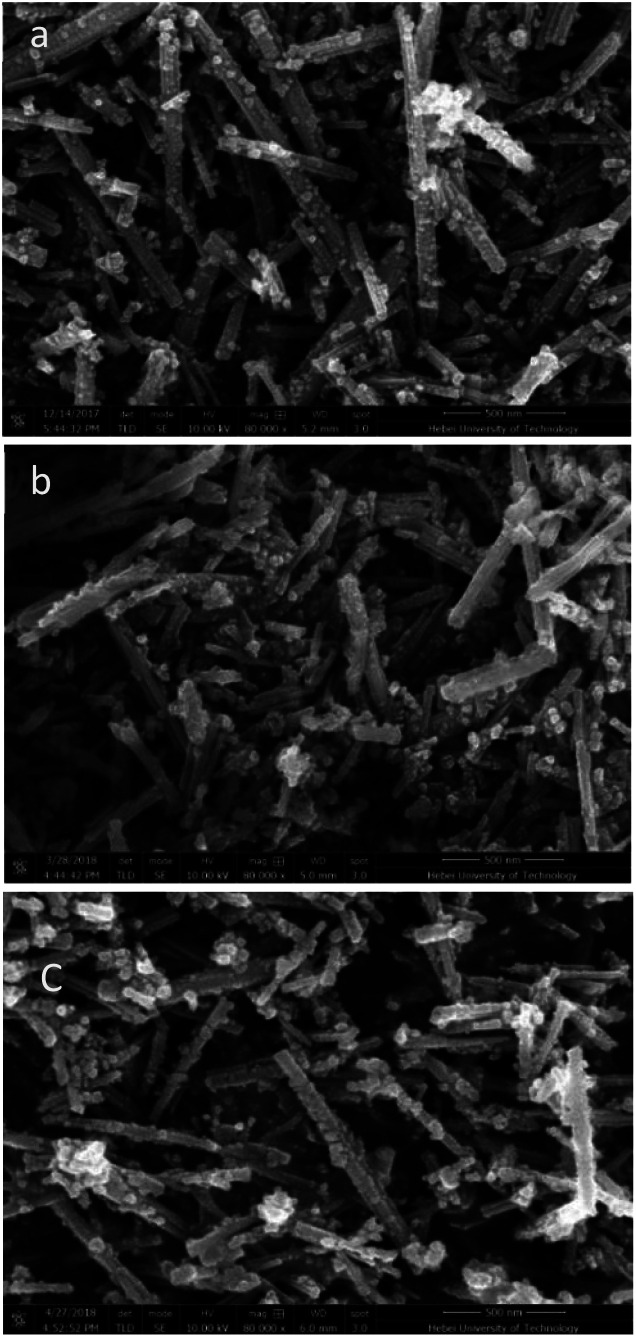
SEM images of (a) CeO_2_-r, (b) Pd/CeO_2_-r, and (c) recovered Pd/CeO_2_-r.

**Fig. 3:**
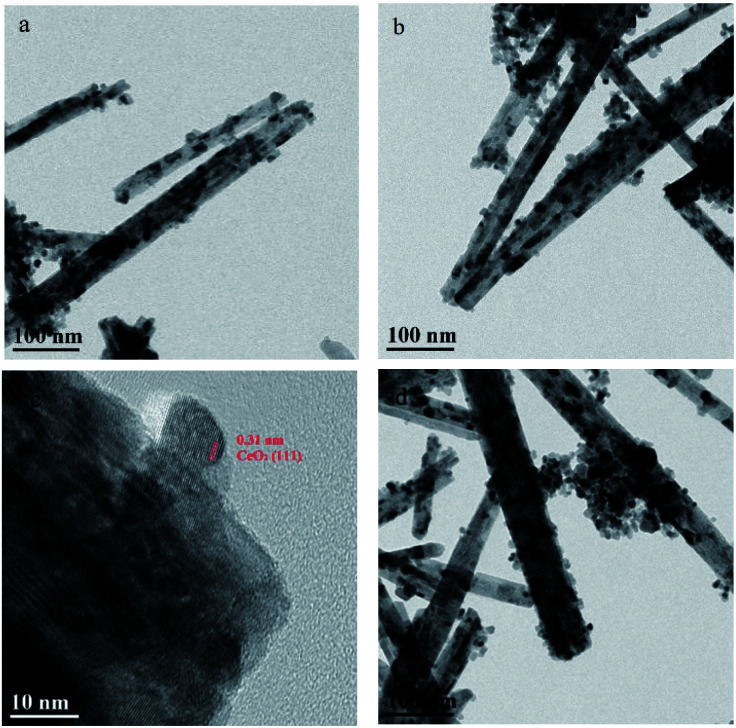
TEM images of (a) CeO_2_-r, (b and c) Pd/CeO_2_-r, and (d) recovered Pd/CeO_2_-r.

The CeO_2_-r and Pd/CeO_2_-r were characterized using SEM (scanning electron microscopy) and TEM (transmission electron microscopy), and the images are shown in Fig. 2 and 3, respectively. Fig. 2(a) is the SEM image of the CeO_2_-r, which shows that most of the CeO_2_-r has a rod-like structure, with lengths from 300 nm to 2 μm, and diameters from 30 to 100 nm. Additionally, there are some small particles attached to the rods, with widths of less than 50 nm. The Pd/CeO_2_-r catalyst was prepared by loading Pd species on the CeO_2_-r, and its SEM image is shown in Fig. 2(b). It can be seen from the figure that the loading has a small effect on the morphology of the CeO_2_-r. Upon loading, although the rod structure remains dominant, the morphology becomes less regular and the length decreases.

Fig. 3 shows the TEM images of the CeO_2_-r and Pd/CeO_2_-r, and the conclusions are similar to those drawn from the SEM images. Both samples comprise rod-like-shaped CeO_2_, with some small particles. Additionally, no obvious Pd species were observed in the Pd/CeO_2_-r. It is generally believed that most Pd particles on a CeO_2_ support cannot be observed, even using HR-TEM.^28^ This may be because the high electron density of CeO_2_ prevents Pd particles from being observed.^29^ An alternative explanation is that the strong interaction between Pd and CeO_2_ inhibits the growth of Pd particles,^23^ and smaller Pd particles cannot be easily observed using TEM. This is consistent with the XRD results.

The textural properties of the CeO_2_-r and Pd/CeO_2_-r were analyzed using N_2_ adsorption–desorption measurements at liquid N_2_ temperature. The results are shown in Table 1. After Pd loading, the surface area and pore volume of the CeO_2_-r decreased markedly, while the average pore size increased. This may be because the Pd species entered the CeO_2_-r pore channels and blocked some of the smaller pores, leaving larger pores open. Wu *et al.*^30^ obtained similar results when loading Pd species on CeO_2_ nanotubes.

**Table 1:** Textural properties and Pd content of CeO_2_-r and Pd/CeO_2_-r

Sample	*A*_BET_ (m^2^ g^−1^)	Pore volume (cm^3^ g^−1^)	Pore diameter (nm)	Pd content^a^ (wt%)
CeO_2_-r	55.2	0.13	9.8	—
Pd/CeO_2_-r	31.6	0.08	14.1	2.0

a Determined by ICP-OES.

### 3.2 Oxidative carbonylation of GL over the Pd/CeO_2_-r

The oxidative carbonylation of GL over the Pd/CeO_2_-r to produce GLC was investigated, and the reaction conditions, including the reaction temperature, pressure, catalyst loading, CO/O_2_ ratio, and reaction time, were optimized.

#### 3.2.1 Effect of reaction temperature

**Fig. 4:**
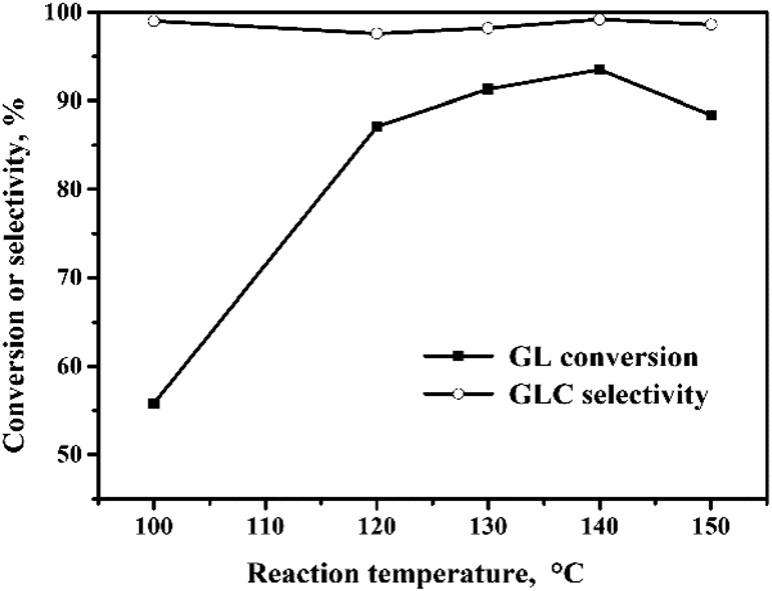
Effect of reaction temperature on the oxidative carbonylation of glycerol. Reaction conditions: GL (25 mmol), Pd : GL = 1 : 2210 (molar ratio), NaI (I^−^/Pd = 10 : 1 in molar ratio), DMA (15 mL), CO (3.3 MPa), O_2_ (1.7 MPa), 100 min.

The influence of the reaction temperature on the OCG reaction over the Pd/CeO_2_-r catalyst is shown in Fig. 4. The GL conversion was 55% when the reaction temperature was 100 °C. The GL conversion increased markedly when the temperature was increased, and reached 93% at 140 °C. Further increase of the reaction temperature resulted in a small decrease of the GL conversion. The Pd/CeO_2_-r-catalyzed OCG reaction is a gas–liquid–solid multiphase reaction, and the solid catalyst is located in the liquid-phase solvent. The reactants, GL, CO and O_2_, are dissolved in the solvent at first, and then react under the action of the catalyst. The solubilities of CO and O_2_ decrease as the reaction temperature is increased. Therefore, from this perspective, high temperature is unfavorable for the reaction. When the reaction temperature is too high, the effect of solubility reduction is more obvious, and the reaction performance and GL conversion are reduced.^19^ In the temperature range investigated in this study, the GLC selectivity was maintained above 98%.

#### 3.2.2 Effect of reaction pressure

**Fig. 5:**
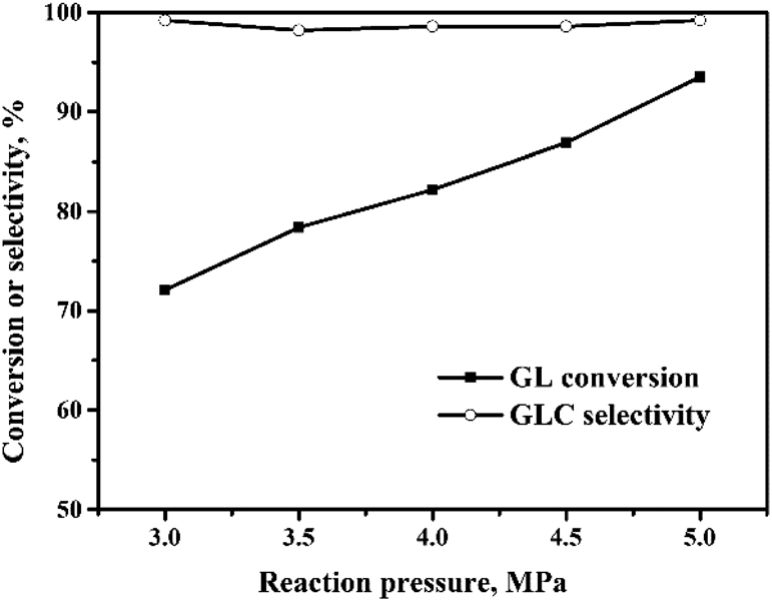
Effect of reaction pressure on the oxidative carbonylation of glycerol. Reaction conditions: GL (25 mmol), Pd : GL = 1 : 2210 (molar ratio), NaI (I^−^/Pd = 10 : 1 in molar ratio), DMA (15 mL), CO : O_2_ = 2 : 1, 100 min, 140 °C.

The reaction pressure is one of the most important factors affecting the OCG reaction. The effect of the pressure was studied, and the results are shown in Fig. 5. GL conversion increased linearly with reaction pressure. When the reaction pressure was increased from 3.0 MPa to 5.0 MPa, the GL conversion increased from 72% to 93%. This is because the OCG reaction is a reaction in which the gas volume is reduced, and so increasing the pressure is favorable for the reaction. Additionally, a high pressure improves the solubility of CO and O_2_ in the solvent. This can also accelerate the reaction and improve the GL conversion. All the GLC selectivities were higher than 98% and were less affected by pressure than the conversion.

#### 3.2.3 Effect of reaction time

**Fig. 6:**
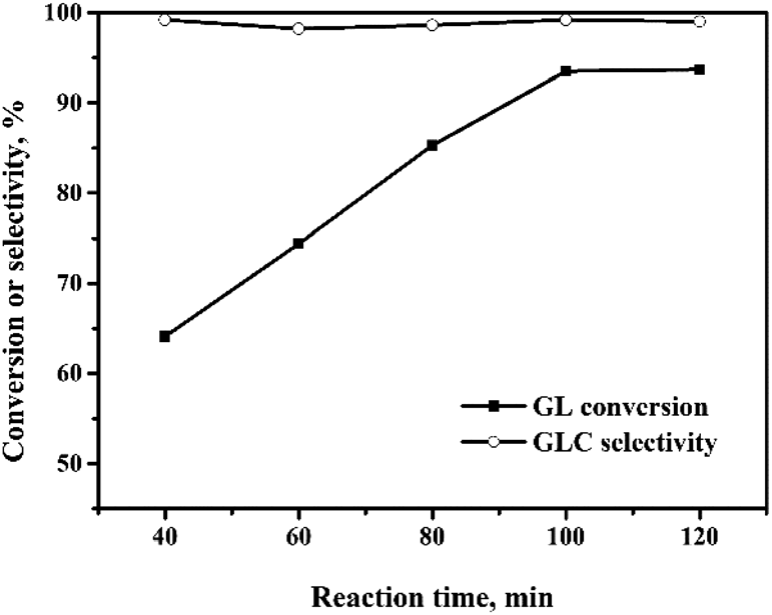
Effect of reaction time on the oxidative carbonylation of glycerol. Reaction conditions: GL (25 mmol), Pd : GL = 1 : 2210 (molar ratio), NaI (I^−^/Pd = 10 : 1 in molar ratio), DMA (15 mL), CO (3.3 MPa), O_2_ (1.7 MPa), 140 °C.

The effect of the reaction time on the OCG reaction over the Pd/CeO_2_-r catalyst is shown in Fig. 6. As the reaction time is increased, the GL conversion increases gradually, reaching 93% at 100 minutes with a TOF of 1240 h^−1^. Upon further increase of the reaction time, the GL conversion changes little. Within the time range of study, the GLC selectivity remained at a high level (not less than 98%).

#### 3.2.4 Effect of CO/O_2_ ratio

**Fig. 7:**
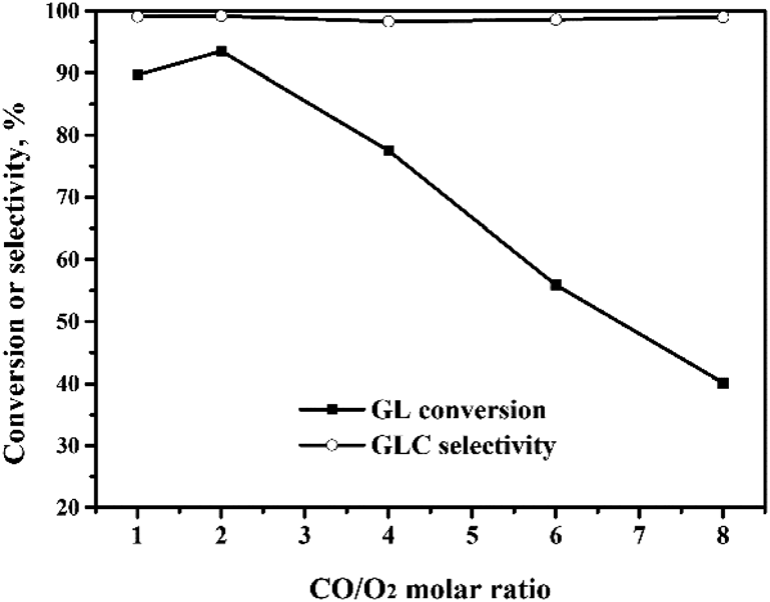
Effect of the CO/O_2_ molar ratio on the oxidative carbonylation of glycerol. Reaction conditions: GL (25 mmol), Pd : GL = 1 : 2210 (molar ratio), NaI (I^−^/Pd = 10 : 1 in molar ratio), DMA (15 mL), 5 MPa, 100 min, 140 °C.

The effect of the CO/O_2_ molar ratio on the OCG reaction was investigated and the results are shown in Fig. 7. The GL conversion increased from 89% to 93% when the CO/O_2_ molar ratio was increased from 1 : 1 to 2 : 1. Upon further increase of the CO/O_2_ molar ratio to 8 : 1, the GL conversion decreased quickly to 40%. Meanwhile, the GLC selectivity changed very little with the CO/O_2_ molar ratio. The role of O_2_ is to ensure that the Pd(0) ↔ Pd(ii) cycle occurs, so that the OCG reaction catalyzed by the Pd species is a catalytic reaction. When the CO/O_2_ ratio is increased, the amount of O_2_ is decreased, which means that the *in situ* oxidation regeneration process, Pd(0) → Pd(ii), is hindered.^21^ This means that the catalyst activity and the GL conversion decrease.

#### 3.2.5 Effect of amount of catalyst

**Fig. 8:**
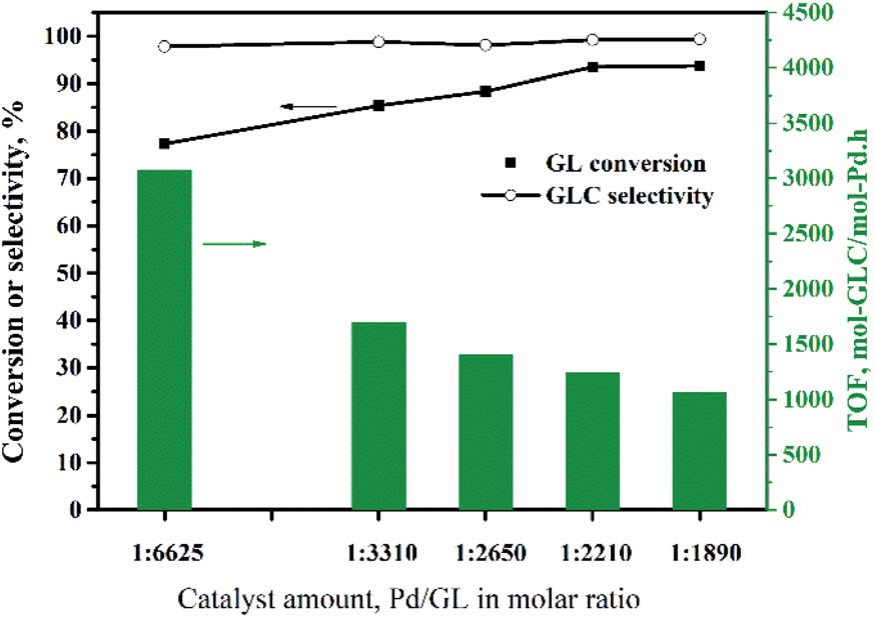
Effect of the amount of Pd/CeO_2_-r on the oxidative carbonylation of glycerol. Reaction conditions: GL (25 mmol), NaI (I^−^/Pd = 10 : 1 molar ratio), DMA (15 mL), CO (3.3 MPa), O_2_ (1.7 MPa), 100 min, 140 °C.

Fig. 8 shows the influence of the amount of Pd/CeO_2_-r catalyst on the OCG reaction. The GL conversion increases gradually as the amount of catalyst increases, reaching a maximum of 93% at a Pd : GL molar ratio of 1 : 2210. Increasing the amount of catalyst further did not improve the GL conversion. Although increasing the amount of catalyst is beneficial to the reaction, too much solid catalyst affects the stirring efficiency and increases the mass transfer resistance. This has negative effects on the reaction. In other words, there is an optimal amount of catalyst. Additionally, the TOF decreased as the amount of catalyst was increased, and was 1240 h^−1^ at the maximum GL conversion, which was higher than those reported for most catalysts for this reaction (Table 2), except the Pd@PQP-NHC catalyst from the latest reports.^8^ For the smallest amount of catalyst used in this experiment, the GL conversion was 77%, and the TOF was 3075 h^−1^, which is the highest value observed in this experiment.

**Table 2:** Comparison of the activities of different Pd-catalysts for oxidative carbonylation of glycerol

Catalyst system	Conversion (%)	Selectivity (%)	TOF (h^−1^)	Ref.
Pd/CeO_2_-r	93	98	1240	This work
Pd/CeO_2_-r	77	99	3075	This work
PdCl_2_(phen)/KI	92	99	184	17
PdCl_2_(phen)@Y	95	98	317	18
Pd/C	82.2	99	900	19
Pd@PQP-NHC	96	99	1901	8

### 3.3 Catalyst stability

**Fig. 9:**
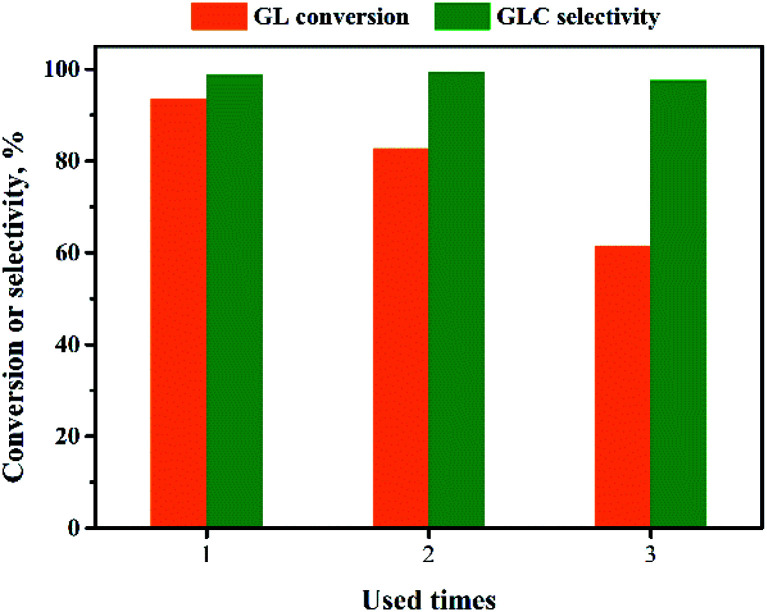
Reusability of Pd/CeO_2_-r.

The reusability of the Pd/CeO_2_-r catalyst was examined. After the reaction, the catalyst was recovered by centrifugation, washed thoroughly with ethanol, and dried at 80 °C for 12 h. It was then re-evaluated for the OCG reaction to produce GLC; the results are shown in Fig. 9. The more times the catalyst had been reused, the lower the GL conversion. The GL conversion was 61% when the catalyst was used for the third time.

To clarify the cause of the catalyst deactivation, the recovered Pd/CeO_2_-r catalyst was characterized using XRD, and the results are shown in Fig. 1(c). In addition to the diffraction peaks assigned to CeO_2_, a new diffraction peak is present at 40.15°. This corresponds to the (111) crystal plane of metallic Pd. This might be present as a result of the aggregation and growth of Pd particles during the reaction. The aggregated Pd would reduce the contact between the reactants and the active centers, thus reducing the activity of the catalyst.

The morphology of the recovered Pd/CeO_2_-r catalyst was observed using SEM and TEM, and the results are shown in Fig. 2(c) and 3(d), respectively. The images show that the morphologies of the catalyst before and after the reaction had no obvious differences, and the rod-like shape was maintained.

The Pd content in the Pd/CeO_2_-r catalyst was determined using ICP-OES (inductively coupled plasma optical emission spectrometry). The results showed that the Pd content was 2.0 wt% and 1.1 wt% in the fresh and recovered Pd/CeO_2_-r catalyst, respectively. Pd leaching during the reaction was also a cause of catalyst deactivation. Wang *et al.*^19^ studied the Pd/C catalyzed OCG reaction. They found that with the aid of NaI, Pd species first dissolved in solvents, and after a period of time, were deposited on the support, with less leaching. They also confirmed that the dissolved Pd species were active in the reaction. The leaching of Pd in our work was much higher than that reported by Wang *et al.*^19^ This may be because the CeO_2_-r support had a smaller specific surface area (31.6 m^2^ g^−1^) than that of the Pd/C used by Wang *et al.* (662.7 m^2^ g^−1^). A smaller specific surface area would not favor the re-deposition of Pd species.

**Fig. 10:**
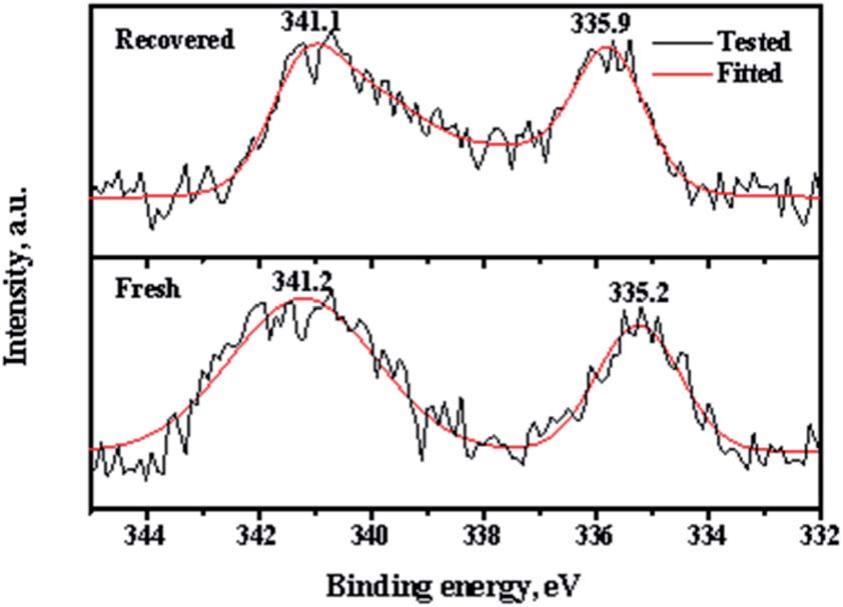
Pd 3d XPS spectra of fresh and recovered Pd/CeO_2_-r.

XPS (X-ray photoelectron spectroscopy) measurements of the fresh and recovered Pd/CeO_2_-r catalysts were used to determine the Pd state, and the results are shown in Fig. 10. There are two peaks in the spectrum of the fresh Pd/CeO_2_-r with binding energies of 341.2 eV and 335.2 eV, which correspond to the Pd 3d_3/2_ and Pd 3d_5/2_ states, respectively. These peaks are assigned to zero valent Pd species.^31^ This indicates that almost all the Pd species were reduced by NaBH_4_. After the reaction, the chemical state of the Pd on the recovered catalyst changed significantly. The binding energies of the Pd 3d peaks increased and the peaks became wider and asymmetric, which indicates the existence of Pd species with high valence. Wang *et al.*^19^ believed that some Pd(0) was oxidized to PdI_2_ during the OCG reaction. However, the change of the Pd valence was not the main reason for the Pd/CeO_2_-r catalyst deactivation, because Pd(ii) is also active in the reaction.^8,19^ To demonstrate this, the PdCl_2_/CeO_2_-r catalyst, without reduction by NaBH_4_, was prepared and evaluated for the OCG reaction. Under the same conditions as those used for the highest conversion with the Pd/CeO_2_-r catalyst, the GL conversion was 82% and the GLC selectivity was 98% over the PdCl_2_/CeO_2_-r catalyst. This indicates that the activity of Pd(ii) was slightly lower than that of Pd(0). Therefore, the main reason for the deactivation of the Pd/CeO_2_-r is the leaching of Pd species.

### 3.4 Proposed mechanism for the OCG reaction over Pd/CeO_2_-r

**Scheme 2:**
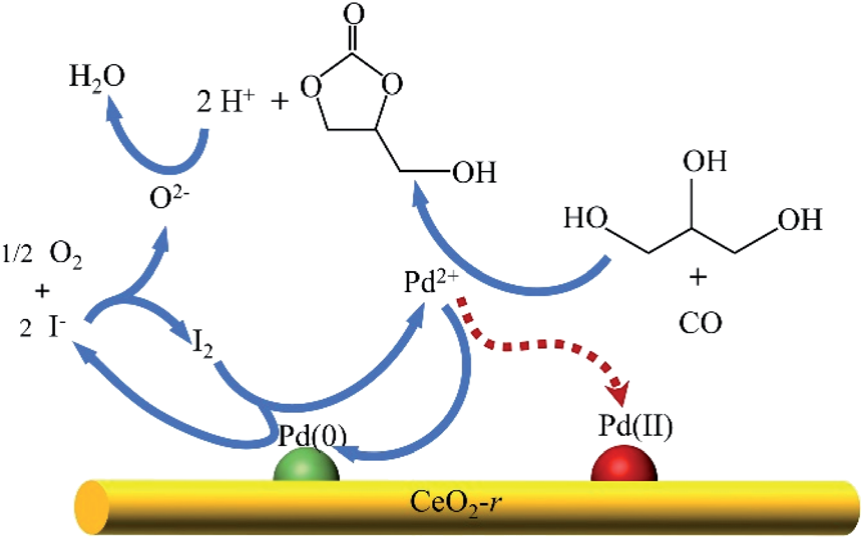
Proposed mechanism for the oxidative carbonylation of glycerol over the Pd/CeO_2_-r catalyst.

Based on the current understanding of the mechanism of Pd-catalyzed oxidative carbonylation^19,23,32^ and the experimental results in this paper, we propose a reaction mechanism for glycerol oxidative carbonylation over the Pd/CeO_2_-r catalyst, which is shown in Scheme 2. In this catalytic cycle, I^−^ first reacts with O_2_ to form I_2_, and then Pd(0) is transformed into Pd^2+^ under the action of I_2_. Pd^2+^ is the active center for the OCG reaction. GLC is then formed from GL and CO, catalyzed by Pd^2+^, and Pd^2+^ is reduced to Pd(0) at the same time. Additionally, some Pd^2+^ is deposited on the CeO_2_-r support in the form of Pd(ii) species. Finally, H^+^ and O^2−^, generated in the previous steps, react to form water to complete the catalytic cycle. Notably, the Pd(ii) species redeposited on the support are also active in the OCG reaction – this is not indicated in the mechanism diagram. In addition, CeO_2_-r support can also promote the Pd-catalyzed OCG reaction. Oxygen can be adsorbed on the oxygen vacancy of CeO_2_-r surface and transformed into lattice oxygen O^2−^; at the same time, Ce^3+^ is oxidized to Ce^4+^, while Ce^4+^ can promote the conversion of Pd(0) generated in the reaction to Pd(ii), thus regenerating the activity.

To understand the heterogeneity of the Pd/CeO_2_-r catalyst, a hot filtration test was carried out under the optimized conditions (GL (25 mmol), Pd : GL = 1 : 2210 (molar ratio), NaI (I^−^/Pd = 10 : 1 in molar ratio), DMA (15 mL), CO (3.3 MPa), O_2_ (1.7 MPa), 140 °C). At first, the reaction was allowed to proceed for 30 min (GL conversion: 51%), after which the catalyst was separated carefully. And the rest of the liquid phase was refilled into the autoclave, which was then pressurized with CO and O_2_, and the reaction continued for another 70 min. The final GL conversion was 72%. The results show that, on the one hand, homogeneous Pd species do have catalytic effect on OCG reaction; on the other hand, the activity of homogeneous Pd is inferior to that of heterogeneous Pd/CeO_2_-r Catalysts, which is due to the promotion of CeO_2_-r support. That is to say, homogeneous and heterogeneous catalysis exist simultaneously in Pd catalyzed OCG reaction.

## 4 Conclusion

CeO_2_ with a rod-like structure (CeO_2_-r) was prepared *via* a hydrothermal method, using P123 as a morphology control agent. It was then used as a support for the Pd/CeO_2_-r catalyst for the oxidative carbonylation of glycerol to produce glycerol carbonate. The Pd/CeO_2_-r catalyst was characterized and evaluated. The optimized reaction conditions were: glycerol (25 mmol), Pd : glycerol = 1 : 2210 (molar ratio), NaI (I^−^/Pd molar ratio = 10 : 1), DMA (15 mL), CO (3.3 MPa), O_2_ (1.7 MPa), 100 min, and 140 °C. Under the optimized reaction conditions, the glycerol conversion and glycerol carbonate selectivity were 93% and 98%, respectively, and the TOF was 1240 h^−1^. When the amount of catalyst was reduced so that the Pd : glycerol molar ratio was 1 : 6625, the TOF was as high as 3075 h^−1^, and the glycerol conversion and glycerol carbonate selectivity were 77% and 99%, respectively. As the number of times the Pd/CeO_2_-r catalyst was reused was increased, the activity of the catalyst gradually decreased. Through characterization, we found that the leaching of active Pd and the growth of Pd particles were the main causes of the deactivation.

## Author contributions

Ziyan Wang, Shuo Guo: performed all the experiments. Zhimiao Wang and Fang Li: written the manuscript and analyzed the experimental data. Yanji Wang: designed the experiments, Wei Xue: conceived the research idea, designed the experiments, interpreted the data, and co-wrote the paper, recruit of funding.

## Conflicts of interest

There are no conflicts to declare.

## Supplementary Material
